# Rehabilitation of Nose following Chemical Burn Using CAD/CAM Made Substructure for Implant Retained Nasal Prosthesis: A Clinical Report

**DOI:** 10.1155/2017/2784606

**Published:** 2017-06-18

**Authors:** Saurabh Chaturvedi, Tushar Bhagat, A. K. Verma, Vishwanath Gurumurthy, Mariyam Ali, Preeti Vadhwani, Mudita Chaturvedi

**Affiliations:** ^1^Department of Prosthetic Dentistry, College of Dentistry, King Khalid University, Abha, Saudi Arabia; ^2^College of Dentistry, Prince Sattam Bin Abdulaziz University, Al-Kharj, Saudi Arabia; ^3^Department of Prosthodontics, Career Post Graduate Institute of Dental Sciences, Lucknow, Uttar Pradesh, India; ^4^Department of Dental Technology, College of Applied Sciences, King Khalid University, Abha, Saudi Arabia; ^5^Department of Prosthodontics, Buddha Institute of Dental Sciences, Patna, Bihar, India; ^6^Department of Oral Pathology, Career Post Graduate Institute of Dental Sciences, Lucknow, Uttar Pradesh, India

## Abstract

Insufficient knowledge of medical chemicals and their improper use have destructive effects. Accidental exposure to chemicals on facial tissue may result in large facial defect. For ages the tradition of piercing nose is common but improper use of unknown chemical for piercing has deleterious effect. Mostly rhinectomy defects are acquired caused by trauma or malignant diseases. Prosthetic rehabilitation is the preferred treatment of choice for any large rhinectomy defects as medical and surgical interventions are ineffective in developing esthetics. Main concern with the prosthesis for such defects is retention. This article describes rehabilitation of a patient with large size nasal defect created by chemical burn in childhood during piercing. Implant retained customized silicone nasal prosthesis was fabricated using simple O-ring attachments and innovative modified polyamide acrylic resin substructure acting as skeleton.

## 1. Introduction

Traditions are the most important part of human civilization but indecorously following these traditions results in deleterious effects. Nose piercing is one of such common customs which is followed for ages. But insufficient knowledge of medical chemicals and their improper use for preparing the nasal tissues for piercing may result in irreversible effects.

The nose is the central part of the face and constitutes the most prominent projection in facial geometry. Corrosive chemical contact with nasal skin will typically lead to tissue damage with the severity of the skin damage depending upon the chemical's strength, quantity applied, duration of contact, degree of skin penetration, and the chemical's mechanism of action. Following chemical exposure, if a strong acid had been used, it usually tended to react with the tissue with which it came into contact by protein coagulation change and penetration to the deep soft tissue base of the skin [1]. If proper medical care is not taken this burn may result in complete deformity of maxillofacial tissues and malignancies.

Each year, a substantial amount of individuals suffers from maxillofacial defects because of trauma, malignant disease, and/or congenital deformity. Amongst them, rhinectomy defects are generally acquired and are caused by benign or malignant neoplasms. Malignancies of the nasal vestibule are rare and account for only 9% of all cancers of the nasal cavity. Squamous cell carcinoma is most common of all tumors of the nasal vestibule [2–4]. Various treatment options available are surgery, radiation therapy, chemotherapy, cryotherapy, immunotherapy, and cytotoxic treatment used alone or in combination [5, 6]. But most of these options leave behind a defect area which needs appropriate rehabilitation either surgically or prosthetically. However, age, general medical condition of the patient, anatomic complexity, possibility of recurrence, large size of defect, complexity of the surgical procedure, and the patient's refusal for surgery result in prosthetic rehabilitation as treatment of choice [4, 7].

Prosthetic rehabilitation has several advantages over the surgical in terms of regular tissue site observation by patient himself, esthetic enhancement, technical simplicity, repeatability, and inexpensive care. This mode of rehabilitation is reliable but in the past the prosthesis had to be attached to the spectacles or stuck on; this was plagued by problems of instability and tissue compatibility [8].

Tjellstrom et al. were the first to use the modified titanium fixtures in the cranial skeleton in 1976 [9, 10]. Reported use of Branemark implants for nasal reconstruction is limited but Sugar and Beumer in two-center study placed 33 implants for nasal reconstruction [11]. Similarly Flood and Russell reported their experience with 30 implants in 14 cases of partial or total rhinectomy followed by early reconstruction with implant retained nasal prosthesis [12]. Thus osseointegrated implants have been used for improving the support and retention. In recent past, many biomaterials and techniques have been used in the fabrication of nasal prostheses. Out of all, silicones proved to be the preferred material. But silicone itself does not adhere to implants; it requires a substructure; CAD/CAM technology associated with rapid prototyping helps in developing of this substructure in highly precise manner, as described by Ciocca et al. [13].

This case report describes rehabilitation of a large size nasal defect caused by chemical burn progressing to complete destruction of nose. An implant retained silicone nasal prosthesis was fabricated utilizing a simple O-ring attachment on a framework of polyamide resin made by CAD/CAM procedure, thus restoring the patient's well-being and improving the quality of life. The retention system used in this case for the prosthesis superstructure was “Implant Based Retention System with O-ring Attachments.” The adhesive was advised to the patient for providing the better camouflage effect and merging the margins more accurately with the adjacent facial surface.

## 2. Case Presentation

**Figure 1:**
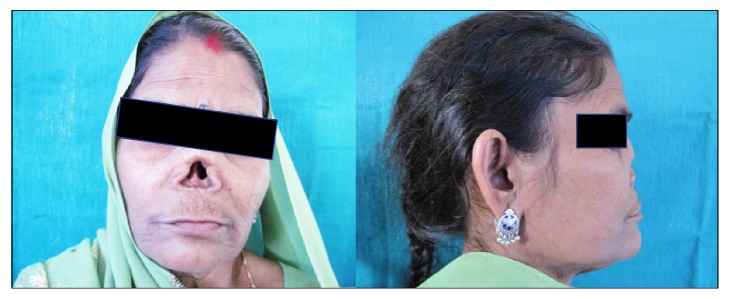
Patient's face with large size nasal defect (frontal and profile view).

A 55-year-old woman with large size nasal defect (Figure 1) was referred to the Department of Prosthodontics, Career Post Graduate Institute of Dental Sciences & Hospital, Lucknow, Uttar Pradesh, India, for a possible prosthetic rehabilitation. Since her childhood, she was like this and apparently did not seek any treatment for the same. She used to cover her face with a piece of cloth and had nasal twang in her voice. She was very shy and silent and only after due encouragement and reassurance she gave her consent for treatment.

History revealed that in name of tradition her parents took her for nose piercing to a rural agent where a chemical was applied to her nose and piercing was done. After that she suffered from some type of infection which starts with some pustule and ulcer and slowly it starts dissolving her nasal tissues. There was bad smell and little pain. As narrated by her relatives that to escape the social trauma her parents did not seek medical care and left her to the destiny. This results in slow and gradual disintegration of nasal tissue including bony skeleton also leaving behind large rhinectomy defects.

On examination, the patient was completely healthy, nasal site of interest appeared normal with healthy skin and mucosal coverage. There was no inflammation, abnormal discharge, or pain on palpation. The apex, right and left alae, lower half of dorsum of nose, and septal cartilages were absent. The mucosa over the anterior part of nasal floor and the remaining tissue margin of the defect was mobile. The mobility of the upper lip was also noted that could potentially compromise the stability of the prosthesis. The maxillary and mandibular arches were partially edentulous. The maxillary anterior teeth were missing and after implant insertion the removable partial denture was used to replace the missing teeth.

**Figure 2:**
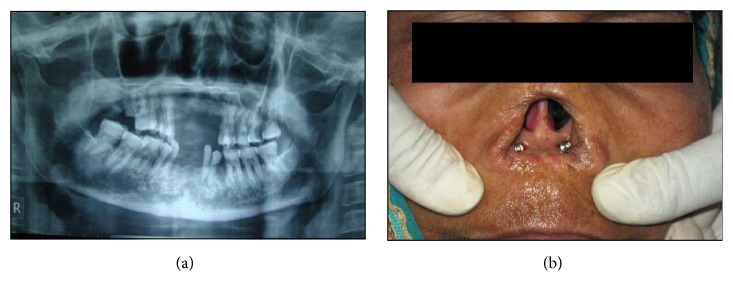
(a) Preoperative panoramic radiograph showing partially edentulous maxillary and mandibular arches. (b) Patient's face with O-ring post over implants in anterior nasal floor.

After thorough evaluation implant supported silicone nasal prosthesis with O-ring attachments was planned for rehabilitation. Presurgical planning included plane radiographs (panoramic radiograph (Figure 2(a)), lateral cephalographs, maxillary occlusal view, and PNS view) of the region and reformatted computed tomogram (CT) for assessment of the bone condition, any pathology and determination of implant size and position. Patient was requested for frontal and profile views of the nose prior to the existing condition but unfortunately only a single frontal photograph was provided as reference.

After facial moulage preparation, special tray with autopolymerising acrylic resin (DPI Self-Cure; Dental Products of India, Ltd.) was made for definitive impression and implant positions were identified. On the basis of clinical and radiographic findings anterior nasal floor (ANF) was selected as site for implant placement.

Planning and insertion of implants in the nasal floor is a complicated procedure when the patient has natural teeth in the anterior portion of the maxilla because of the risk of damaging the roots of the natural teeth during the surgical procedure while inserting the implants. When digitally planning the implants according to the technique of Van der Meer et al. [14], the implants can be safely inserted in the nasal floor of dentate subjects.

In our case, the maxillary anterior teeth were missing so the level of complication was little less comparatively. We used various radiographs and Cone Beam Computer Tomography to determine the bone level and angulation of the premaxillary area, associated anatomical structures, and placed implants. It has been suggested that dental fixtures may be placed in the alpha sites to retain nasofacial prostheses. Alpha sites are 6 mm or greater in axial bone volume. The most common areas of the facial skeleton having this volume of bone are the anterior maxilla through the nasal fossa (floor of the nose) and the zygoma [15].

Initially two-stage implant placement was decided, following the techniques described by Lundgren et al. [16], Stanley and Olsen [17], and Parel and Tjellstrom [18]. But later, to avoid stage II surgery, it was decided to place the healing caps in place of cover screw, over the implants in stage I itself. Two conventional implants of 3.3 mm diameter and 10 mm length (Adin Dental Implant Systems Ltd.) were placed in the ANF parallel to palate. Surgical templates were used to assist in positioning the implants. Implants were allowed to heal for 6 months. After healing period a soft tissue with thickness of 2–4 mm and width of 2-3 mm was developed at the implant site. Now the healing caps were removed and the selected O-ring posts were screwed over the implants (Figure 2(b)).

Utilizing special tray, definitive impression of the defect site was made using light body polysulfide impression (Aquasil Ultra Lv, Dentsply, Germany) material. The impression was boxed and poured in type IV gypsum product (Kalabhai Karson, Mumbai, India). The resulting master cast was sprayed with an oil free release agent (Silicone Spray, Dentsply Caulk) to facilitate the removal of wax pattern and prevent the wax from soaking into the gypsum cast. The prosthesis was sculpted in wax with the esthetic contours developed. Remaining anatomical landmarks and previous photograph provided by patient were used as reference.

**Figure 3:**
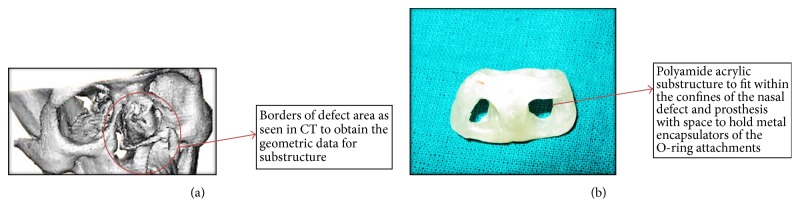
(a) CT image of defect area. (b) Polyamide acrylic substructure.

For fabrication of substructure, mold was made by CAD/CAM. Patient's nasal defect area was scanned by CT to obtain the geometric data (Figures 3(a) and 3(b)).

**Figure 4:**
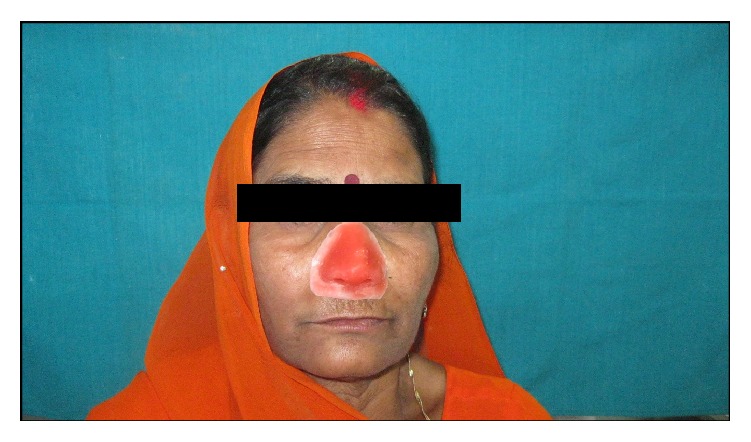
Evaluation of wax pattern on patient.

These data were further processed by a CAD system to generate both positive and negative replicas of the normal substructure for nose, and the resultant data were input into a CAM system (Spectrum 510, Z Corporation) for the fabrication of the resin models. This substructure was designed to fit within the confines of the nasal prosthesis and to hold the metal encapsulators of the O-ring attachments at a further stage. Following this try-on of wax pattern (Figure 4) and acrylic substructure try-in were done and verified for shape, extension, air way, and esthetics. Patient's approval and suggestions were taken into consideration.

**Figure 5:**
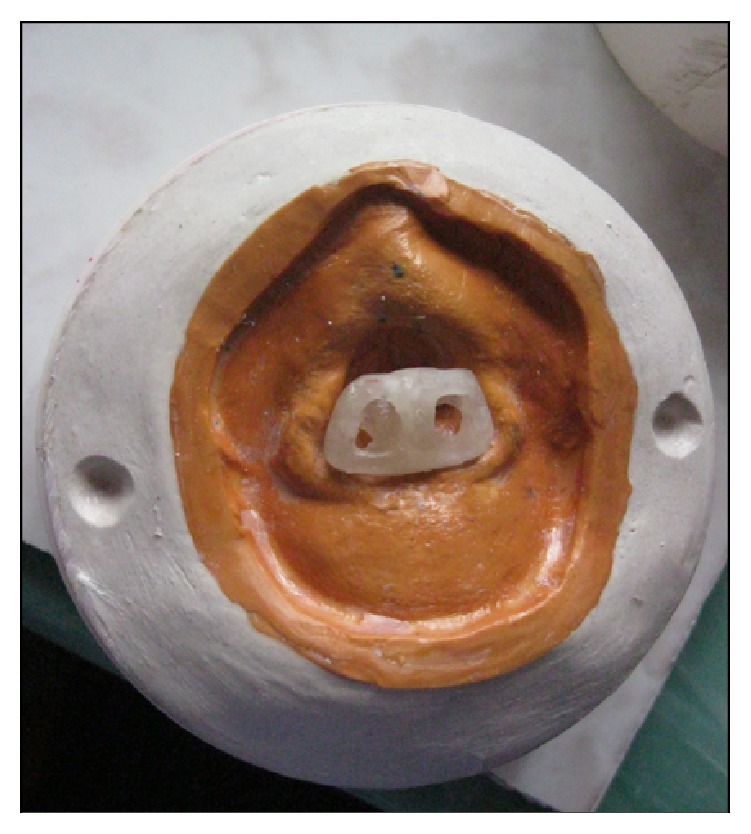
Acrylic resin substructure on dewaxed master cast.

On completion of try-on step, the wax sculpture was sealed into place on the master cast. Dental plaster was injected into the areas of the nares to maintain the air way passage and the entire wax pattern was invested. After the wax boil-out procedure, the acrylic substructure was positioned on the master cast (Figure 5).

The medical grade room temperature vulcanizing silicone (Dow Corning Corp, Midland, Michigan) was mixed and packed in the mold after evaluating color from the patient. Layer wise packing was done to produce a multilayered intrinsically colored prosthesis. After packing, the mold was clamped under pressure at room temperature for 24 hours. Following complete curing, the prosthesis was retrieved from the flask and trimmed and finished carefully with the help of scissors and silicone finishing burs. Limited flash was preserved around the borders of the nasal prosthesis to facilitate transition of the borders to the adjacent tissue. The definitive prosthesis was checked critically for the silicone tags, color, shape, and incorporation of acrylic substructure and its exposure. To achieve the bonding between polyamide substructure and the silicone both chemical and mechanical methods were used, to be sure that debonding between two would not take place very early. The substructure was made hollow (as seen in Figure 5 and care was taken to maintain the space for metal encapsulator during packing of silicone) so that the silicone engages it from all around and adhesives were also applied on its surface to bond with silicone.

**Figure 6:**
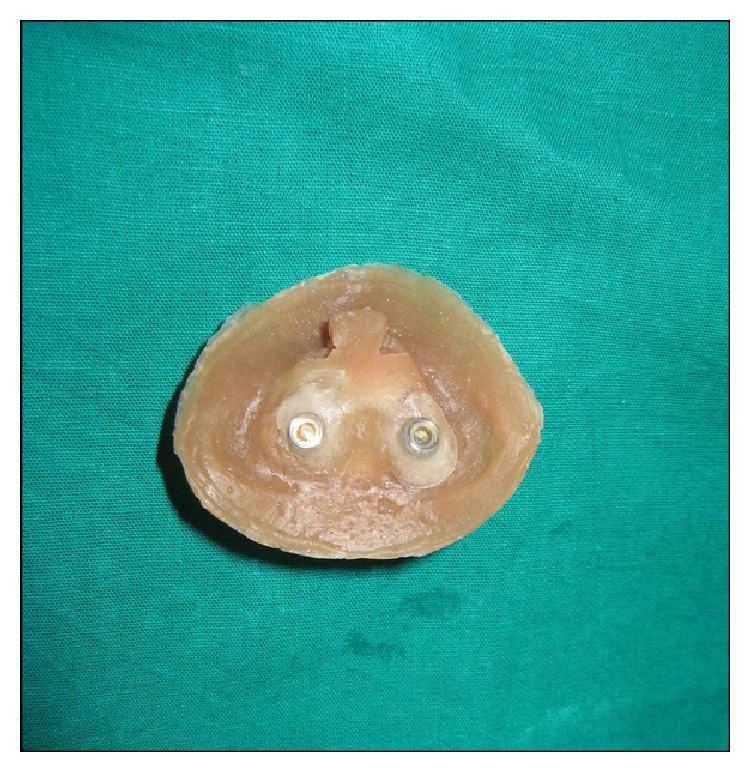
Definitive nasal prosthesis with metal encapsulator of O-ring attachment incorporated in resin substructure.

The silicone material had filled the space for the metal encapsulators of O-ring attachment, so it was cut off from the area and acrylic was roughened. Now on the O-ring posts over implants, the O-ring with 30–40 hardness in a Shore A scale was seated along with the corresponding metal encapsulator. The previously made polyamide acrylic substructure which was incorporated in the nasal prosthesis was repositioned over the implants in its accurate position. The prosthesis was removed and evaluated for the incorporation of metal encapsulator with O-ring in acrylic resin substructure (Figure 6).

**Figure 7:**
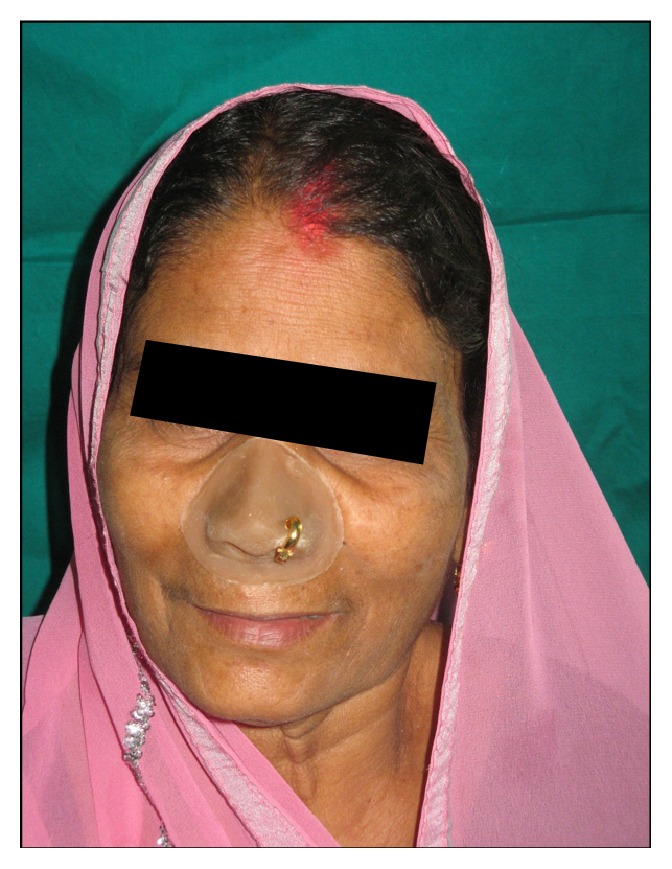
Definitive nasal prosthesis on patient.

**Figure 8:**
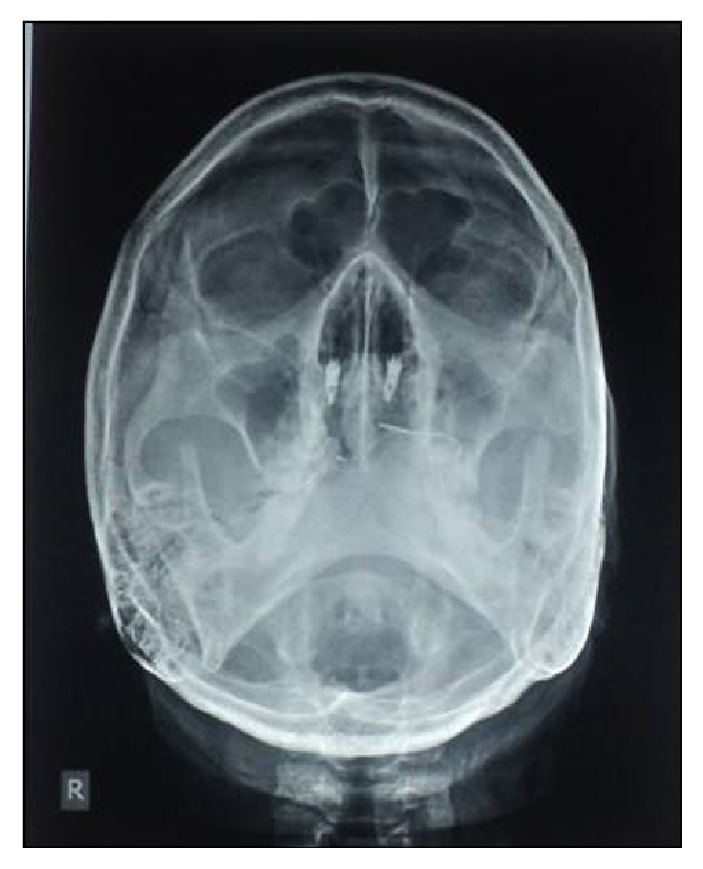
Postoperative PNS water's view radiograph showing implant in position.

Now the completed nasal prosthesis was delivered to the patient (Figure 7). The patient was instructed for prosthesis placement, removal, and application of the medical grade skin adhesive (Pros-Aide; ADM Tronics Inc., Northvale, NJ) at the borders. Home care and maintenance included mechanical debridement with a soft toothbrush and proxy brush, irrigation with warn saline water and soap, and/or 2% hydrogen peroxide. The color match was satisfactory and appreciated by patient and her husband. Elevation in the spirit of patient was seen in her eyes and happiness of her husband could be judged by the action that he bought a new nose ring to his wife. Patient was observed regularly for 6 months which was found satisfactory and verified by radiograph (Figure 8).

## 3. Discussion

Chemical assault and its misuse have been reported in many countries. The reported facial lesions are varying in dimension from 34 to 76% of body surface area, and the severity depended to some extent upon different cultures and causes. Medical and surgical interventions of large nasal defects to achieve esthetics are very challenging as discussed by Chou et al. [1]. Prosthetic management of nasal defects resulting from trauma, burn, or surgery has been well-documented. A definitive nasal prosthesis can reestablish esthetic form and anatomic contours for this type of midfacial defect, often more effectively than by surgical reconstruction. However, in the past, many patients have not been satisfied with prosthetic reconstruction mainly because of inadequate retention and secondary dermatitis [17]. Osseointegrated implant has changed this scenario completely as in this case report. Use of implants and their success for nasal prosthesis were justified in several previous studies [12, 18–22]. In the presented case intraoral implants were used because of their added length, as the use of short implants correlates with a higher incidence of failure [12, 23, 24]. Both cortical plates of nasal floor and palatal plate were engaged to gain initial stability. ANF is an excellent implant site, as described by Nishimura et al. [25] and Granstrom et al. [26], for placement of conventional intraoral implants because of sufficient bone availability and excellent vasculature. This site also facilitated hygiene access. Two implants were sufficient in number for providing adequate retention and stability.

For retaining the prosthesis O-ring attachments were used which provided a stress breaking design over bar attachment which results in unfavorable leverages over the implants resulting in bone loss (Davis B et al.'s stress distributions of implants used for retention of maxillary obturators. Paper presented at Seoul International Congress on Maxillofacial Prosthetics, Seoul, 1996). The added advantages of the O-rings were easy replacement of worn-out rings and range of hard O-rings for needed amount of retention. Along with this there was unavailability of magnets and macroolder bar system in our area and financial constrains of the patient. Use of bars may limit access for performing hygiene procedures and make it difficult to insert and remove the prosthesis. When magnets were used, all eventually demonstrated corrosion; most magnets exhibited signs of corrosion within 6 months. The discoloration secondary to the corrosion eventually required remaking the facial prosthesis.

New CAD/CAM technologies have led to improved approaches for the construction of nasal prostheses substructure. Polyamide acrylic substructure from rapid prototyping method resulted in providing exact shape, structure, and adherence with the implants. The most important advantage is the preciseness. The exact dimensions of substructure which confined accurately in the defect area with sufficient space for silicone would be made possible by CAD/CAM procedure which uses polyamide acrylic. Polyamide acrylic has several advantages such as an excellent esthetic characteristic, low water sorption and solubility, adequate strength, low toxicity, easy repair, and a simple molding processing technique. Secondly the residual monomer content in conventional acrylic sometimes is irritating to the patient and it leaches out of prosthesis resulting in shrinkage of prosthesis. The chances of discoloration are always more in acrylic substructure of conventional acrylic compared to polyamide. Although, scientists have reported the automation of impression making for nasal prostheses and diagnostic wax-up, substructure design by CAD and printing by means of rapid prototyping machines, try-in automation and the elimination of stone molds, and optimized surface roughness due to changes in the surface of the prototyped mold [23, 24, 26–28]. But, overall cost factor imposes limitation. When a nasal prosthesis has to be stabilized in place through implants via O-ring, long-term follow-up of the connection system is very important. The main features of this connection should be esthetic invisibility and sufficient mechanical support for the prosthesis during function. In this protocol, a novel substructure design of polyamide acrylic instead of metal framework for a nasal prosthesis was created.

Various biomaterials such as polymethyl methacrylate, polyvinyl chloride, polyurethane, and silicone are available for fabrication of nasal prosthesis. But silicone is the material of choice as it is flexible and easy to merge with adjacent tissues and provide natural like appearance as in this presented case [29]. But the limited service of maxillofacial prostheses is usually a consequence of deterioration of the elastomer and color change under environmental exposure to sunlight and change in temperature, humidity and hand contact during cleaning, and adhesive use on a daily basis (Chen et al. [30], 1981, and Hanson et al. [31], 1983)

Also the silicone materials fall short of an ideal maxillofacial prosthetic material because of their poor adheophilic property, polishing problems, low tear resistance, and microbial growth-promoting characteristics. The most critical properties of an ideal maxillofacial prosthetic material are esthetics, durability, and accuracy of processing [32]. Patients are concerned with the durability and esthetics of the prosthesis. A prosthesis must be durable, esthetic, and color stable. The limited service of facial prostheses is a result of the rapid degradation of the elastomer and its color instability. The wearing time for facial prostheses averages from 3 months to 1 year. Deterioration is mainly caused by environmental exposure to ultraviolet (UV) light, air pollution, and changes in humidity and temperature. Handling the prosthesis during cleaning and the application of adhesives and cosmetic additives may also alter the physical properties and color stability of the material [30, 31, 33, 34].

Finally the success of the prosthesis is judged by the satisfaction expressed by the patient and her relatives which was surely achieved in this case.

## Additional Points

*Summary*. In the name of tradition use of harmful chemicals may result in life long taboo. As in this case report the use of chemical of nose piercing results in large nasal defect causing retention issues during fabrication of maxillofacial prosthesis. This case report describes that the predictable biomechanical retention of nasal prostheses can now be achieved using osseointegrated implants. The silicones made implant retained nasal prostheses are tissues compatible and simple in design and easy to place and can be worn independently of spectacles. This not only enhances the esthetics but also provides confidence to the patient to face the world.
